# Brain-computer interface with somatosensory feedback improves functional recovery from severe hemiplegia due to chronic stroke

**DOI:** 10.3389/fneng.2014.00019

**Published:** 2014-07-07

**Authors:** Takashi Ono, Keiichiro Shindo, Kimiko Kawashima, Naoki Ota, Mari Ito, Tetsuo Ota, Masahiko Mukaino, Toshiyuki Fujiwara, Akio Kimura, Meigen Liu, Junichi Ushiba

**Affiliations:** ^1^Department of Biosciences and Informatics, School of Fundamental Science and Technology, Graduate School of Keio UniversityKanagawa, Japan; ^2^Department of Rehabilitation Medicine, Keio University School of MedicineTokyo, Japan; ^3^Department of Rehabilitation Medicine, Asahikawa Medical University HospitalAsahikawa, Japan

**Keywords:** brain-computer interface rehabilitation, motor imagery, somatosensory feedback, visual feedback

## Abstract

Recent studies have shown that scalp electroencephalogram (EEG) based brain-computer interface (BCI) has a great potential for motor rehabilitation in stroke patients with severe hemiplegia. However, key elements in BCI architecture for functional recovery has yet to be clear. We in this study focused on the type of feedback to the patients, which is given contingently to their motor-related EEG in a BCI context. The efficacy of visual and somatosensory feedbacks was compared by a two-group study with the chronic stroke patients who are suffering with severe motor hemiplegia. Twelve patients were asked an attempt of finger opening in the affected side repeatedly, and the event-related desynchronization (ERD) in EEG of alpha and beta rhythms was monitored over bilateral parietal regions. Six patients were received a simple visual feedback in which the hand open/grasp picture on screen was animated at eye level, following significant ERD. Six patients were received a somatosensory feedback in which the motor-driven orthosis was triggered to extend the paralyzed fingers from 90 to 50°. All the participants received 1-h BCI treatment with 12–20 training days. After the training period, while no changes in clinical scores and electromyographic (EMG) activity were observed in visual feedback group after training, voluntary EMG activity was newly observed in the affected finger extensors in four cases and the clinical score of upper limb function in the affected side was also improved in three participants in somatosensory feedback group. Although the present study was conducted with a limited number of patients, these results imply that BCI training with somatosensory feedback could be more effective for rehabilitation than with visual feedback. This pilot trial positively encouraged further clinical BCI research using a controlled design.

## Introduction

Stroke leads to a rapid loss of brain function through a disturbance in the blood supply to the brain and usually causes hemiparesis. Data from an earlier study suggest that practicing or observing movements that are highly similar to normal movements helps to improve motor functions (Ertelt et al., 2007; Garrison et al., 2010; Arya et al., 2011). Experience-based plasticity mechanisms, that involve the relative re-weighing of synaptic inputs, are constantly shaping network organization and are more likely driven by the formation and elimination of dendritic spines (Johnston, 2004; Carmichael, 2006; Murphy and Corbett, 2009). Some animal studies suggest that such plasticity occurs at both the peri-lesion and remote areas (Nudo, 2006). The results of several randomized, controlled, trials have indicated that the intensive practice of important motor tasks, while constraining the non-paretic limb, can substantially improve upper limb function in individuals whose movements have been mildly impaired by stroke (Grotta et al., 2004; Mark et al., 2006; Taub et al., 2006; Lin et al., 2010). In the case of moderate impairment, assisted voluntary movement with functional electrical stimulation through surface electrodes is effective in improving finger and wrist motor functions (Peckham et al., 1980; Kimberley et al., 2004).

Recently, electroencephalogram (EEG)-based brain-computer interface (BCI) has been regarded as a new rehabilitation technique for patients with severe impairment after stroke, who cannot use the other above-mentioned rehabilitation strategies owing to a lack of volitional muscle activity (Buch et al., 2008; Daly et al., 2009). Motor imagery is often used in EEG-based BCI, because it is defined as the mental rehearsal of a motor act without overt movement (Alkadhi et al., 2005). BCI estimates the patients' motor imagery from the amplitude of the arc-shaped waveform on an EEG, or a magnetoencephalogram recorded over the primary sensorimotor cortex (SM1) and translates it into feedback (e.g., visual guidance, electrical stimulation of muscles, or motor-driven orthosis). Imagery, or an actual hand movement, activates the SM1 and rhythmic activity in the alpha and beta band over the hand region results in amplitude attenuation or event-related desynchronization (ERD). This enables movement observation or provides afferent feedback in the BCI, and such feedback is believed to help direct brain reorganization, resulting in some functional recovery from stroke hemiplegia (Daly and Wolpaw, 2008). The prolonged use of this BCI training induces plastic changes in the brain activity of patients with stroke (Rozelle and Budzynski, 1995; Buch et al., 2008) and clinical improvement of upper limb function (Prasad et al., 2009; Caria et al., 2011; Shindo et al., 2011; Ramos-Murguialday et al., 2013; Mukaino et al., 2014)

However, specifications of the BCI paradigm that are needed for functional recovery are as yet unknown. As Daly and Wolpaw speculated, neural plasticity will be guided in different ways depending on the feedback modality. Visual feedback of ongoing SM1 excitability trains patients to produce normal SM1 activity, whereas robotic assistance of paretic movement following SM1 excitation will produce sensory input that induces neural plasticity to restore more normal motor control. To date, different types of feedback have been separately tested in some research groups. Thus, the validation of feedback type and protocol standardization in a BCI rehabilitation context will be beneficial for further research development.

In this paper we compared two different types of feedback (i.e., visual feedback and sensory feedback with robotic movement assistance) contingent to motor-related EEG in stroke patients with chronic hemiplegia with a view toward functional recovery, using the Stroke Impairment Assessment Set (SIAS) which is a known standard scoring test, consisting of 22 subcategories, and has high reliability. BCI settings, except feedback and the task design, were shared between the two paradigms in order to minimize the potential influences of factors such as training intensity, duration, and adaptation to the EEG classification rules. Since such an experiment was first designed as a pilot trial, the experiments were conducted as a group comparison study to minimize participants' burden from an ethical point of view. We note here that data in this BCI paradigm (sensory feedback) was previously reported elsewhere as a preliminary case series study (Shindo et al., 2011). On the other hand, the goal of our study was to compare two different types of feedback. Thus, the same data was used for another research purpose in this article.

## Methods

### Participants

The study group consisted of 12 participants who had had a stroke (three with right and nine with left hemiplegia) and met the following inclusion criteria: (1) the first episode was a subcortical stroke; (2) they had severe upper limb paralysis and a score ≤2 for finger movement on SIAS (see Appendix) (Chino et al., 1994), indicating very clumsy finger movement and absence of isolated individual finger movement; (3) they had no cognitive impairment; and (4) their chronic stroke injury occurred more than 13 weeks prior to the study to ensure that further neurological and clinical recovery were less likely (Nakayama et al., 1994; Jørgensen et al., 1995; Duncan et al., 2000). Detailed clinical information of the 12 participants is shown in Table 1. Twelve participants had little or no detectable surface electromyogram (EMG) activity from the affected extensor digitorum communis (EDC) when they attempted to extend their fingers. All participants provided written informed consent prior to participating in the study.

**Table 1:** **Patient characteristic and clinical evaluation**.

**Participant**	**Age**	**Lesion**	**TFO (month)**	**SIAS**	**Feedback**
1	41	Right putamen	4	1a	Visual
2	84	Right caudate nucleus	4	1b	Visual
3	63	Right corona radiate	7	1c	Visual
4	52	Middle cerebral artery area	31	1a	Visual
5	49	Right putamen	13	1a	Visual
6	65	Right putamen	10	0	Visual
7	47	Right thalamus	23	1a	Somatosensory
8	65	Right corona radiate	155	1a	Somatosensory
9	65	Right corona radiate	25	1a	Somatosensory
10	60	Right internal capsule	51	1a	Somatosensory
11	54	Left putamen	23	1a	Somatosensory
12	46	Left putamen	24	1b	Somatosensory

TFO, time from onset.

### Experimental paradigm

**Figure 1:**
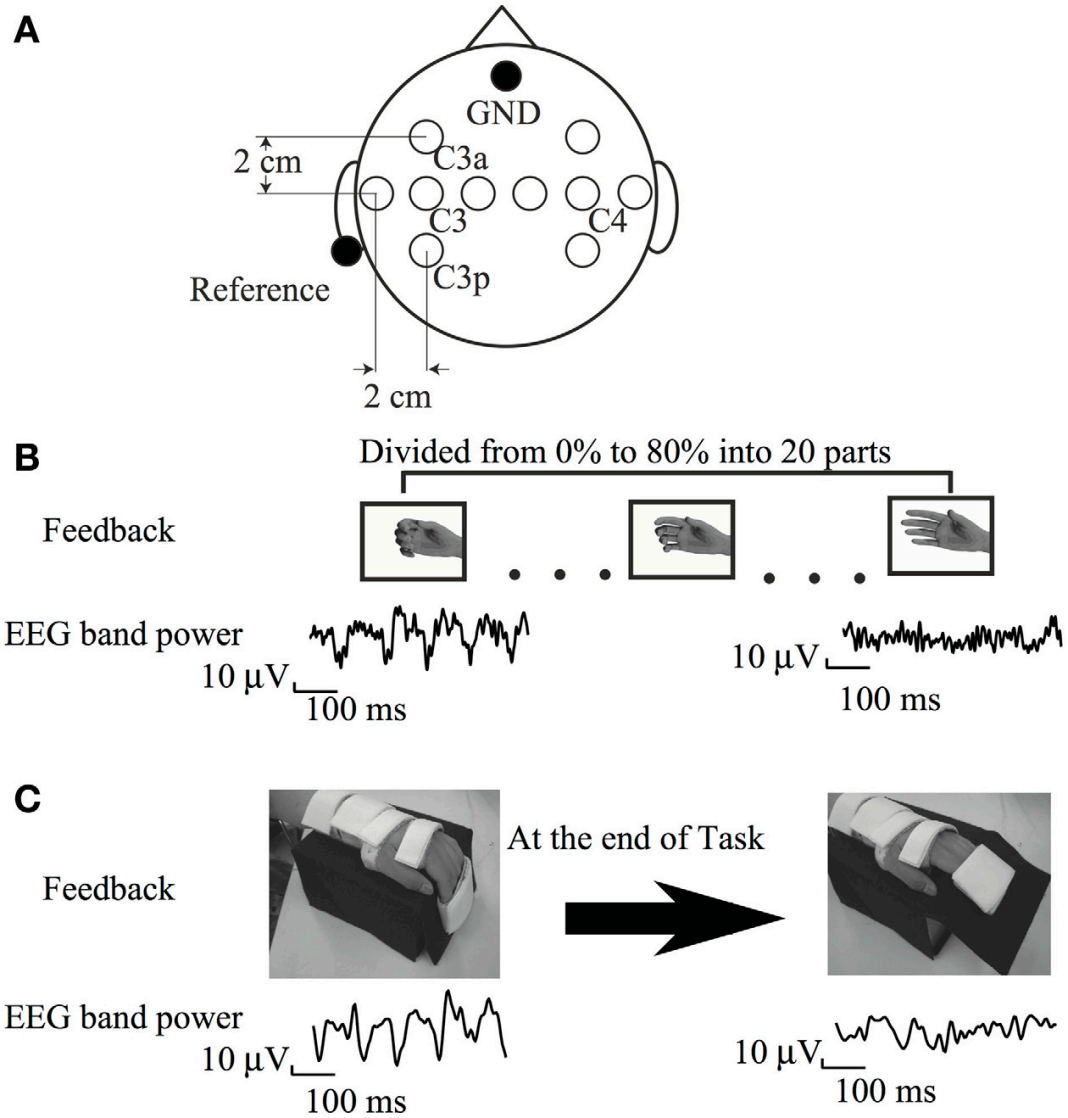
**(A)** Electrode position **(B)** Visual feedback paradigm **(C)** Somatosensory feedback paradigm cited from Shindo et al. (2011), partially revised.

The experimental protocol was conducted in accordance with the Helsinki Declaration and was approved by the ethical committee of Keio University. The experiment consisted of BCI training and brain activity assessment using EEG. The BCI training protocol was similar to that reported previously (Neuper et al., 2009). Participants were seated in a comfortable chair with their arms supported and relaxed on the armrests in pronation. A 15.4-inch computer monitor was placed about 60 cm in front of their eyes. EEG signals were recorded using 10 Ag/AgCl disc electrodes (ϕ = 10 mm) placed on both hemispheres (Figure 1A). The reference electrode was placed at the left auricle. The signals were amplified (g.USBamp; Guger Technologies, Graz, Austria) and digitized (sampling frequency, 256 Hz). The surface EMG was recorded bilaterally from the EDC muscles (high-pass filter 5 Hz; sampling rate 256 Hz). Impedance of EMG electrodes was kept under 10 kOhm.

EEG signals were processed using MATLAB (MathWorks Inc., USA). Firstly, all bipolar combinations were calculated from five electrodes over each hemisphere. Secondly, all EEG trials were visually controlled for artifacts and contaminated trials were discarded (Neuper et al., 2009). EEG spectra were estimated by fast Fourier transformation, using 1-s window lengths, 90% overlap, and a Hanning window. Feedback was generated on the ERD value calculated for predefined participant-specific frequency bands (Pfurtscheller et al., 1997) using the following equation:

$$ERD(f,t)=\frac{R(f)-A(f,t)}{R(f)}\times 100$$

where *A*(*f,t*) is the power spectrum of the EEG at frequency *f* at time *t*, with reference to the onset of the motor task (see *BCI training* below), and *R*(*f*) is the power spectrum of a 1-s epoch of the reference period (2–3 s) in each trial. By using this definition, ERD was expressed as a positive number. The time-frequency map of each bipolar signal was calculated from a 1-s EEG window after every 125 ms. This time-frequency data was used to select the most reactive frequency band and a bipolar montage. If an ERD was not observed at the beginning of BCI training, we used EEG power at a base frequency (9–12 Hz) with a bipolar montage of C3a-C3, which was the best electrode scheme in general (Neuper et al., 2006). A three-factor [time (pre-, post-training), side (damaged, undamaged hemisphere), feedback type (visual, somatosensory)] analysis of variance (ANOVA) for the ERD.

### BCI training session

In this study, there were two feedback groups. Six participants performed BCI training with visual feedback and six participants performed BCI training with somatosensory feedback.

#### Visual feedback

The trial was initiated upon presentation of the word “Relax” on a monitor, and a countdown was presented at the bottom of the monitor to prepare participants to attempt extension of an affected finger. The word and countdown disappeared 5 s later. Six participants received a visual feedback stimulus from the EEG in the form of a picture of the affected hand on the monitor. The ERD value in response to the resulting action of the feedback was determined before training as follows: firstly, participants generally achieved an increase in sensorimotor rhythm during voluntary relaxation and an ERD while imagining maximal finger extension on the paralyzed side. Pictures of the hand with varying degrees of hand movement were then mapped according to ERD magnitude. We prepared 20 pictures depicting different hand positions, ranging from a full-hand grasp to a fully open hand. A hand opening in the picture was associated with increasing ERD (Figure 1B). Pictures of a hand closing were associated with decreasing ERD because the participants' hands were normally positioned in a more grip-like posture during the passive state, caused by spasticity. The ERD was divided into 20 parts from 0 to 80%, and each part was assigned 1 hand picture. The hand picture on the screen then remained stable, and the participants were asked to relax for 5 s. This 15-s trial was repeated for approximately 1 h, and a total of 100 trials were performed. This training was conducted on 5 weekdays for 1 month. The experiment was discontinued for the day if the participant complained of exhaustion. Because some participants complained of exhaustion during multiple sessions, the training time was shortened; however, these participants were asked to perform at least 60 trials on that day.

#### Sensory feedback

The participants had to imagine the paretic hand opening or at rest for 5 s according to the task cue. The height of the cursor reflected the accumulated value of the output of classification of ERD performed every 30 ms since the beginning of the task. Thus, the cursor fluctuated around the baseline if diminution of ERD was not clearly seen. The cursor went down if the diminution of ERD was continuously observed. The gain of cursor movement was within approximately one-tenth of the vertical range of the monitor during the resting phase in the calibration experiment. From the 4th training day, when the cursor reached the lower half on the right edge of the monitor, the motor-driven orthosis was triggered to extend the paralyzed fingers from 90 to 50° (Figure 1C). Each training run consisted of 10 trials, with 5 trials per class, presented in randomized order. Ten training runs were recorded per day, with a total of 100 trials.

#### Outcome assessment

Surface EMG activities of the affected EDC muscle and ERD were compared between the first and last training days. The task was slightly modified from the BCI training paradigm in order to easily perform paretic finger movements. The cursor moved from the left to right over a period of 8 s on the monitor, and the task cue was presented 5 s after the cursor had appeared. Participants were instructed to perform “unaffected hand opening” or “affected hand opening” voluntarily for 3 s. This training run consisted of 10 trials with 5 trials per class, alternately.

In assessing improvement of finger movement impairment, SIAS was used at pre- and post-BCI training. It consists of a scale from 0 to 5, with 0 indicating complete paralysis and 5 no paresis (see Appendix).

## Results

### Neurophysiological changes

**Figure 2:**
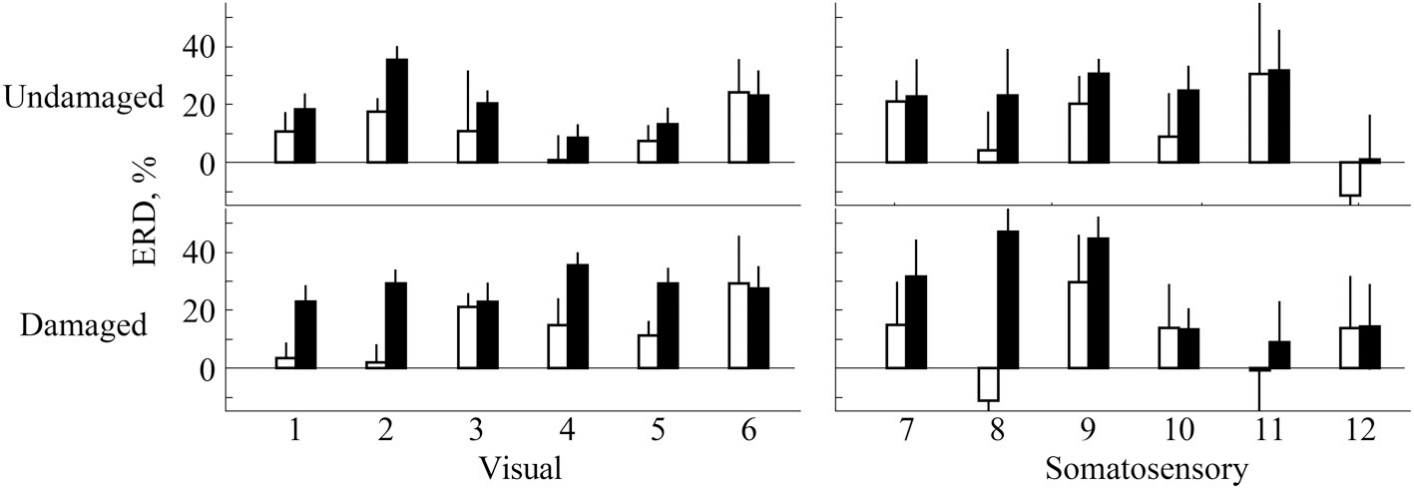
**ERD evaluation over both primary sensorimotor areas**. White bars represent ERD values before training and black bars represent the ERD values after training. Numbers on x axis represent participant numbers.

**Figure 3:**
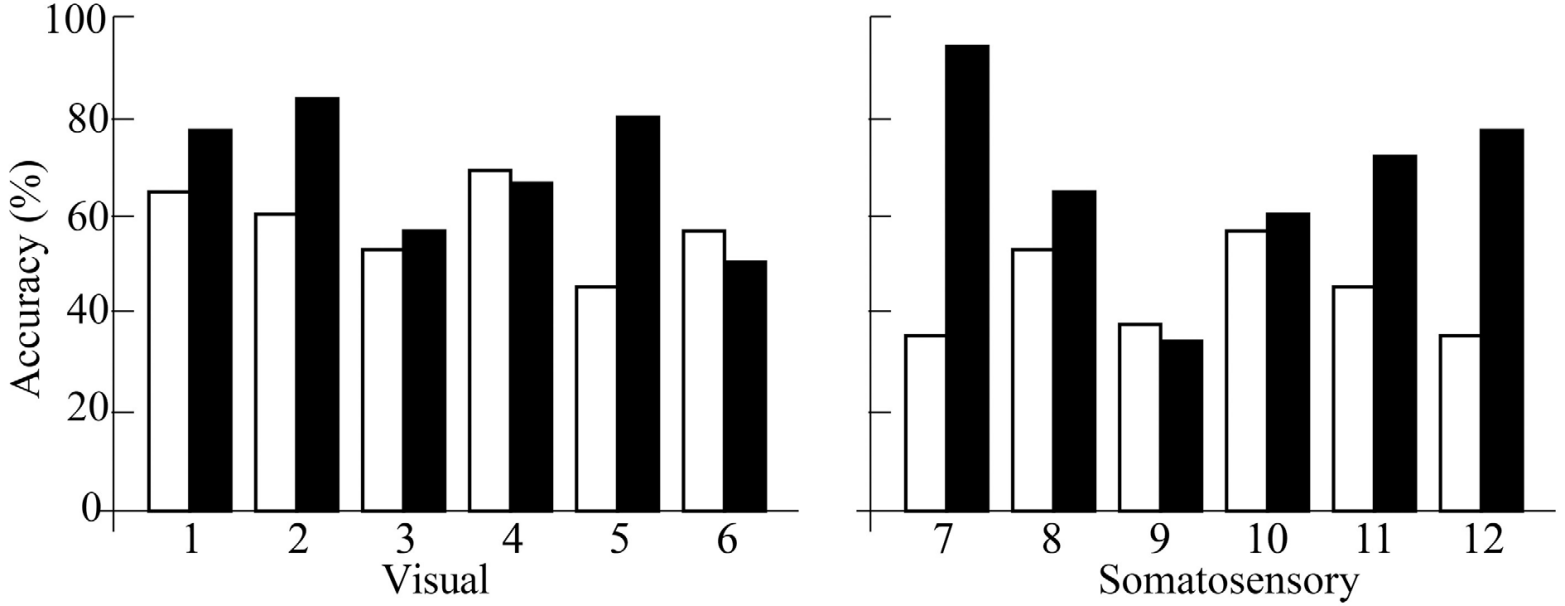
**BCI performance accuracy**. White bars represent ERD values before training and black bars represent the ERD values after training. Numbers on x axis represent participant numbers.

ERD in most participants was detected over both the damaged and undamaged hemispheres, in alpha and/or beta frequency bands throughout the experiment. Figure 2 showed the ERD value before/after trainings in both hemispheres. Statistical evaluation of ERD values revealed significant enhancement over both damaged and undamaged hemispheres after BCI training in participants in both feedback categories (ERD values were shown in Table 2). Three-Way ANOVA showed no significant differences of side and feedback type, but it became significantly greater over both hemispheres (*p* < 0.05). Figure 3 shows BCI performance. BCI performance increased in both feedback groups, while there was no significant difference between feedback groups (*p* < 0.05; Two-Way ANOVA).

**Table 2:** **ERD values of each hemisphere (mean ± *SD* %)**.

	**Visual**		**Somatosensory**	
	**Before**	**After**	**Before**	**After**
Undamaged	12.1 ± 8.3	20.0 ± 9.2	15.9 ± 9.7	22.2 ± 11.1
Damaged	13.6 ± 10.4	27.9 ± 5.0	14.1 ± 9.0	26.3 ± 16.9

**Figure 4:**
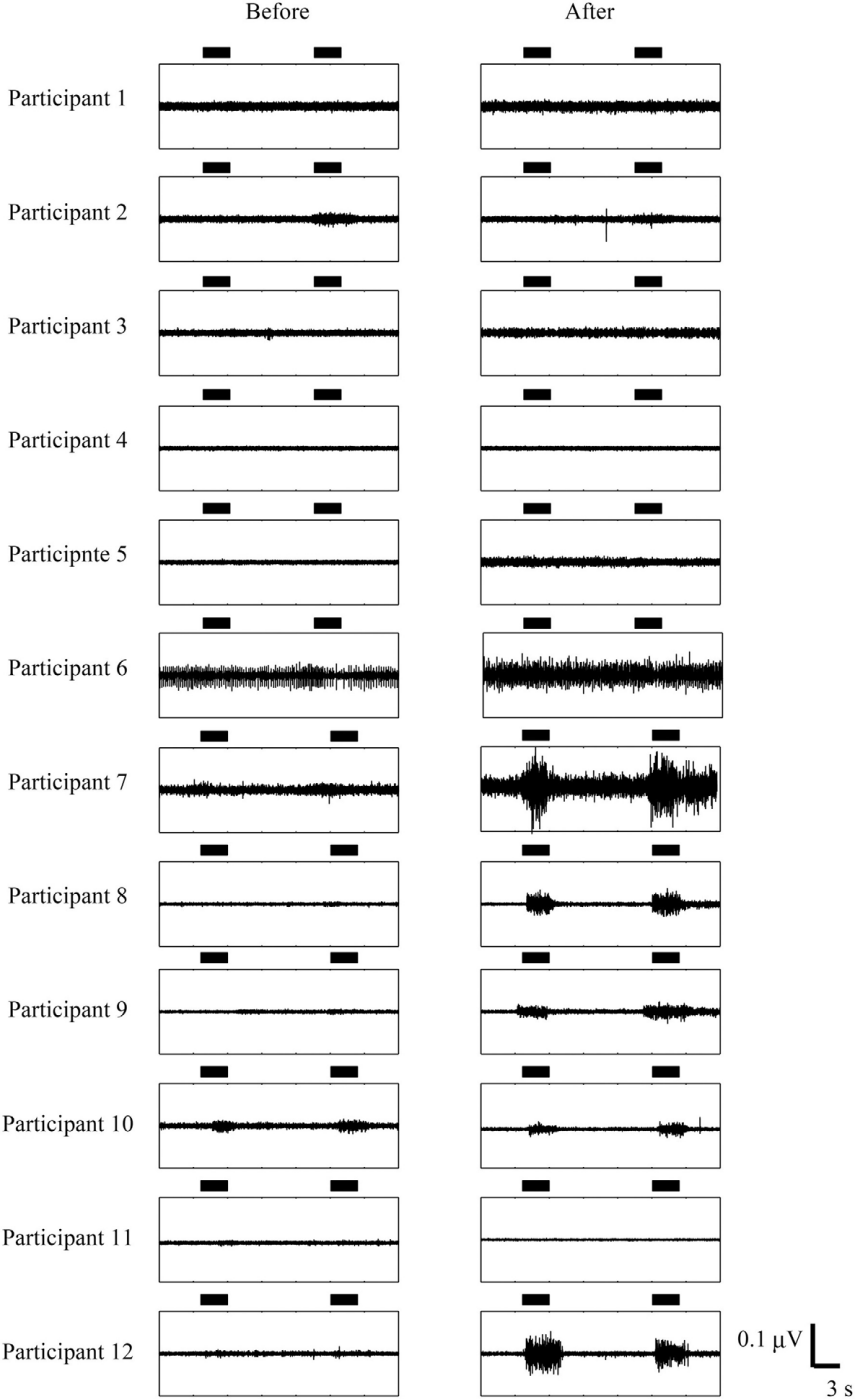
**Comparison of EMG activity before and after BCI**. The horizontal bars represent the period during which participants opened their paralyzed hands. The data from participant 7 to participant 12 are from Shindo et al. (2011). Permission from Foundation for Rehabilitation Information.

Figure 4 showed EMG activities of affected EDC before/after trainings. Four participants in the somatosensory feedback group, who had little or no muscle activity before training, showed EMG activity voluntarily, while no participants in the visual feedback group improved their EMG activity. These results indicated that participants in the sensory feedback group improved in finger function and/or voluntary EMG activity. Note here that the visual feedback group did not show any improvement even when they received a longer training period.

### Clinical behavioral changes

**Figure 5:**
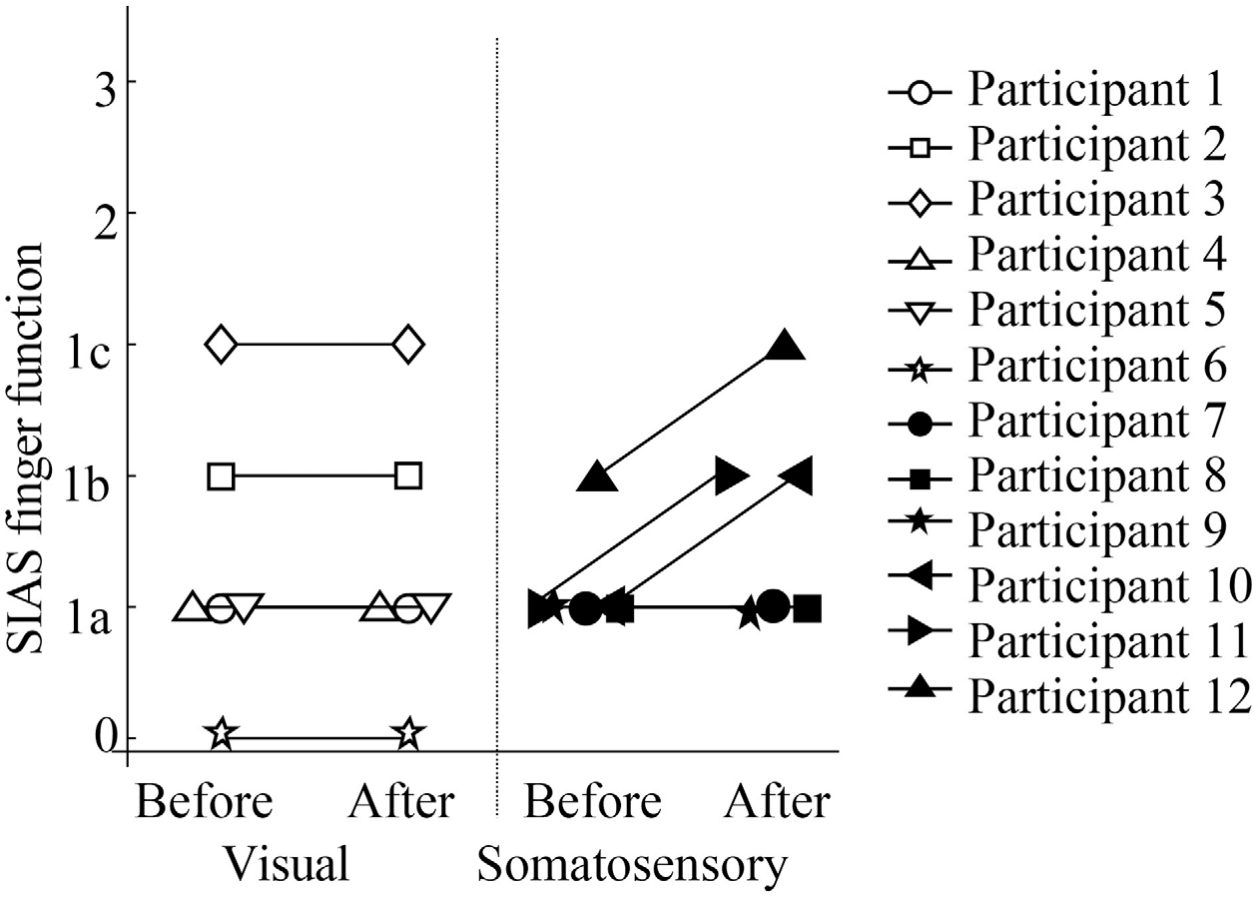
**Stroke Impairment Assessment Set (SIAS) finger function scores**.

Figure 5 showed scores of SIAS finger function test. While no participants in the visual feedback group showed improvement in their finger function, three participants in the sensory feedback group showed improvement in finger function. All participants felt that they could relax more easily, although no participants in the visual feedback group improved on any scores. In addition, participants in the somatosensory feedback group indicated that they became more aware of the use of their paretic upper extremity in daily activities.

## Discussion

These results show that EEG-based BCI training with visual or sensory feedback enhanced ERD following attempted motor activity, but only sensory feedback improved motor function. Though only a limited number of patients participated in the current study, the results of this preliminary study suggest that a randomized controlled trial to complement these results be completed in the future.

### ERD and finger function

Participants in this study learned to increase ERD after training. In addition, BCI performance also increased in both groups. These results follow those of previous studies (Pfurtscheller and Neuper, 2001; Buch et al., 2008; Hwang et al., 2009; Broetz et al., 2010; Hashimoto et al., 2010; Shindo et al., 2011; Cincotti et al., 2012; Mukaino et al., 2014). However, in the visual feedback group, no functional improvement was seen in any participants. From these results, we can say that ERD may not be a direct correlate of functional recovery in finger movement. ERD likely reflects desynchronized neural assembly as a result of the interaction between the thalamic nuclei and cortical areas, that are controlled by the interplay among thalamic relay cells and reticulo-thalamic pathway cells (Steriade and Llinás, 1988; Lopes da Silva, 1991). Desynchronization that is not directly related to motor output is potentially learned by visual feedback BCI.

ERD may reflect SM1 excitability during the relevant motor task (Takemi et al., 2013), thus a proper sensory feedback which engages the participant in the task may facilitate motor reorganization. Moreover, since the nature of neural activity is non-linear, a supplemental neural excitation factor, i.e., timing-dependent cortical excitation by contingent somatosensory feedback to the motor cortex, may promote further excitation of the SM1, resulting in functional recovery. These possibilities could explain why only sensory feedback BCI had a tendency to promote functional recovery in stroke hemiplegia.

### Training interval

Due to a limitation in hospital regulations, visual feedback training was done on 5 weekdays for 1 month and somatosensory feedback training was done once or twice a week for a period of 4–7 months. Of course, the training schedule should be the same between groups, however the results and remarks remain valid, since even intensive (everyday) and longer (1 month) training with visual feedback BCI did not show functional recovery. This suggests that sensory feedback following a motor attempt may be essential for reorganization of motor function. Intensive bodily sensation of the paralyzed limb may also be helpful to regain body awareness (or ownership), which is needed for motor planning. Such a compound effect may make sensory feedback more advantageous that visual feedback BCI

## Conclusion

We performed ERD-regulated motor imagery training in a BCI framework in stroke patients who have chronic, severe, hemiplegia, and observed ERD enhancement. Sensory feedback rather than visual feedback of ERD tended to restore paretic finger movement. These results reveal the importance of peripheral bodily sensation contingent to voluntary excitation of the cortical motor system, which is a key in promoting behavioral improvement. This is a serial case study with clinical limitations. Although the small number of participants, differences in training intervals and duration since stroke are limiting factors, these results provide interesting, positive, data which indicate that a further, large-scale, clinical trial be undertaken, which we expect would support these preliminary insights.

## Funding sources

This study was partially supported by the Strategic Research Program for Brain Sciences (SRPBS) of the Ministry of Education, Culture, Sports, Science, and Technology, Japan.

### Conflict of interest statement

The authors declare that the research was conducted in the absence of any commercial or financial relationships that could be construed as a potential conflict of interest.

## Appendix

### Stroke impairment assessment set (SIAS)

**Table:**

Motor Function (Finger)
Finger test: Individual finger movements are tested. The patient flexes each digit from the thumb to the little finger, in that order, and then extends them from the little finger to the thumb.
0: No voluntary finger movement
1a: Minimal voluntary movement or mass flexion
1b: Mass extension
1c: Minimal individual movement
2: Individual movement of each finger is possible, but flexion or extension is not complete
3: Individual movement of each finger is possible with adequate flexion and extension of the digits; however, the patient carries out the task with severe or moderate clumsiness
4: The patient carries out the task with mild clumsiness
5: The patient carries out the task as smoothly as for the unaffected side
